# Urinary interleukin-18 does not predict acute kidney injury after adult cardiac surgery: a prospective observational cohort study

**DOI:** 10.1186/cc6972

**Published:** 2008-08-01

**Authors:** Michael Haase, Rinaldo Bellomo, David Story, Piers Davenport, Anja Haase-Fielitz

**Affiliations:** 1 Department of Intensive Care, Austin Health, Melbourne, 145 Studley Rd, Heidelberg 3084, Australia; 2 Department of Nephrology and Intensive Care Medicine, Charité University Medicine, 1 Augustenburger Platz, Berlin 13353 Germany; 3 Department of Anaesthesiology, Austin Health, Melbourne, 145 Studley Rd, Heidelberg 3084, Australia; 4 Department of Medicine, Monash Medical Centre, 168 Clayton Rd, Clayton 3168, Melbourne, Australia

## Abstract

**Introduction:**

Urinary interleukin-18 (IL-18) measured during the immediate postoperative period could be a promising predictor of acute kidney injury following adult cardiac surgery.

**Methods:**

In a single-centre prospective observational cohort study, we enrolled 100 adult cardiac surgical patients undergoing cardiopulmonary bypass at a tertiary hospital. We measured the urinary concentration of IL-18 and creatinine preoperatively, on arrival in the intensive care unit, and 24 hours postoperatively. We assessed urinary IL-18 concentration and urinary IL-18/urinary creatinine ratio in relation to the postoperative development of acute kidney injury defined as an increase in serum creatinine of greater than 50% from preoperative to postoperative peak value within 48 hours after surgery.

**Results:**

Twenty patients developed acute kidney injury. On arrival in the intensive care unit and at 24 hours postoperatively, urinary IL-18 (median [interquartile range]) was not different in patients who subsequently developed acute kidney injury compared with those who did not: on arrival in the intensive care unit (168 [717] versus 104 [256] pg/mL; *P *= 0.70) and at 24 hours (195 [483] versus 165 [246] pg/mL; *P *= 0.47). On arrival in the intensive care unit (area under the curve for the receiver operating characteristic curve [AUC-ROCC] 0.53, 95% confidence interval [CI] 0.38 to 0.68; *P *= 0.70) and at 24 hours postoperatively (AUC-ROCC 0.55, 95% CI 0.40 to 0.71; *P *= 0.48), urinary IL-18 was not better than chance in predicting acute kidney injury. All findings were confirmed when urinary IL-18 was adjusted for urinary creatinine. Urinary IL-18 correlated with duration of cardiopulmonary bypass (*P *< 0.001).

**Conclusion:**

In adults, early postoperative measurement of urinary IL-18 appears not to be valuable in identifying patients who develop acute kidney injury after cardiac surgery, but rather represents a nonspecific marker of cardiopulmonary bypass-associated systemic inflammation.

## Introduction

Acute kidney injury (AKI) is a serious and frequent complication of cardiac surgery [1-3]. Cardiac surgery is the second most frequent cause of AKI worldwide among critically ill patients [2]. There is a need for early and specific renal biomarkers that would enable the early prediction of AKI and timely intervention [4]. There is evidence that urinary interleukin-18 (IL-18) – a proinflammatory cytokine released in response to injury to renal tubular epithelial cells and demonstrated in activated macrophages – might act as an earlier biomarker than serum creatinine in predicting AKI in critically ill adult patients [5-8]. However, in the prediction of contrast-induced nephropathy in adult patients, the value of urinary IL-18 is controversial [9,10]. Recently, urinary IL-18 was reported to predict AKI in patients receiving a kidney transplant [11], in critically ill children [12], and in paediatric cardiac surgery [13]. Despite such a promising observation in children, the predictive performance of the measurement of urinary IL-18 immediately after surgery has not yet been assessed in adult cardiac surgical patients undergoing cardiopulmonary bypass (CPB), the majority of patients receiving cardiac surgery. Accordingly, in this prospective observational cohort study, we sought to investigate whether urinary IL-18 could serve as an early predictive renal biomarker in adult patients undergoing cardiac surgery. We hypothesised that urinary IL-18 measured during the immediate postoperative period would be a promising predictor of AKI following adult cardiac surgery.

## Materials and methods

### Patient population

In a prospective observational cohort study, we screened 114 adult patients who underwent cardiac surgery necessitating the use of CPB at a tertiary hospital. We excluded patients undergoing emergency operations (time between hospital admission to operation of less than 24 hours) or an operation without the use of CPB, patients with end-stage renal disease (serum creatinine of greater than 300 μmol/L), kidney transplant patients, and patients under the age of 18. The local institutional review board of the Austin Hospital, Melbourne, approved this investigation and written informed consent was obtained. This study was in compliance with the Declaration of Helsinki. Clinical practice was not changed or modified for the purpose of the study.

### Outcomes

**Figure 1:**
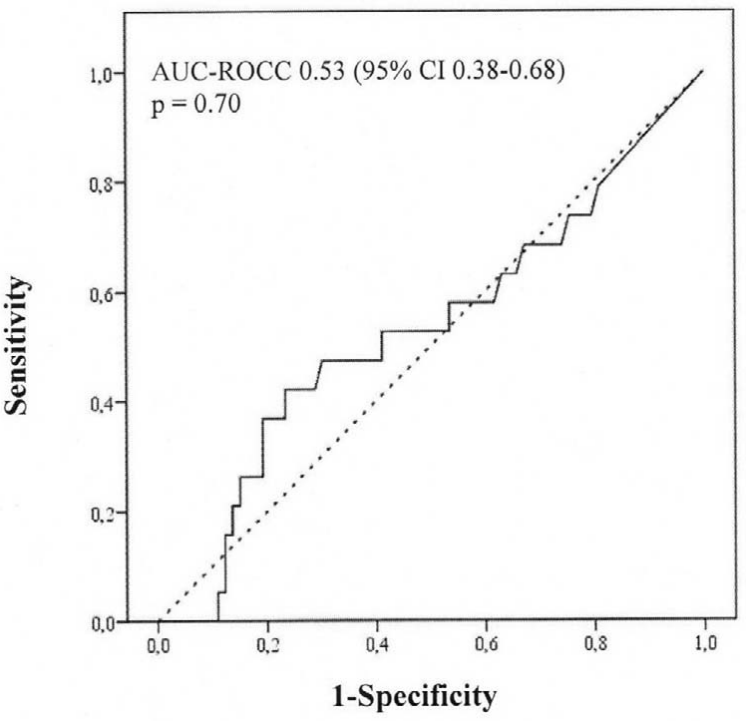
Performance of urinary interleukin-18 on arrival in the intensive care unit as a predictor of acute kidney injury according to an increase in serum creatinine of greater than 50% from preoperative to postoperative peak value within 48 hours after commencement of cardiopulmonary bypass (n = 20). AUC-ROCC, area under the curve for the receiver operating characteristic curve; CI, confidence interval.

**Figure 2:**
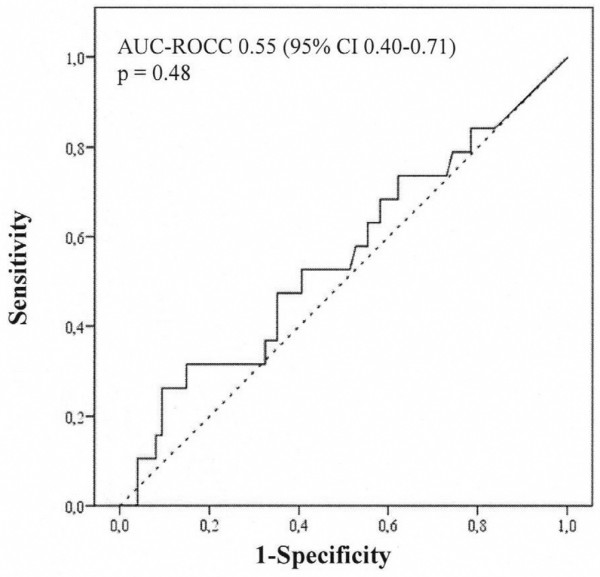
Performance of urinary interleukin-18 at 24 hours after commencement of cardiopulmonary bypass as a predictor of acute kidney injury according to an increase in serum creatinine of greater than 50% from preoperative to postoperative peak value within 48 hours after commencement of cardiopulmonary bypass (n = 20). AUC-ROCC, area under the curve for the receiver operating characteristic curve; CI, confidence interval.

**Figure 3:**
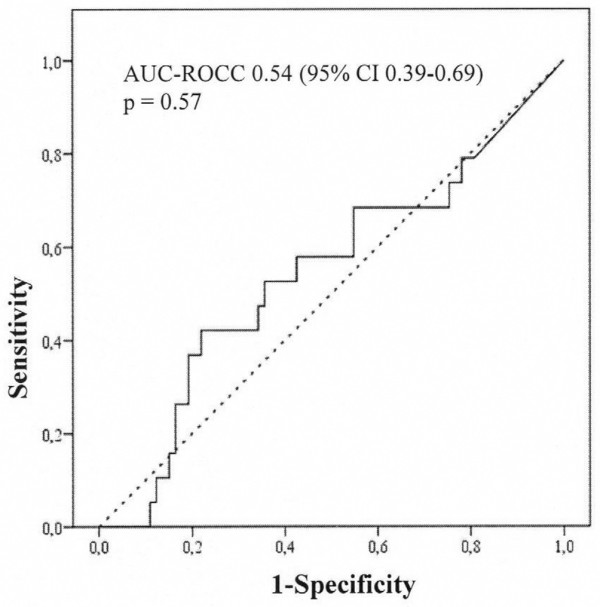
Performance of urinary interleukin-18/urinary creatinine ratio on arrival in the intensive care unit as a predictor of acute kidney injury according to an increase in serum creatinine of greater than 50% from preoperative to postoperative peak value within 48 hours after commencement of cardiopulmonary bypass (n = 20). AUC-ROCC, area under the curve for the receiver operating characteristic curve; CI, confidence interval.

**Figure 4:**
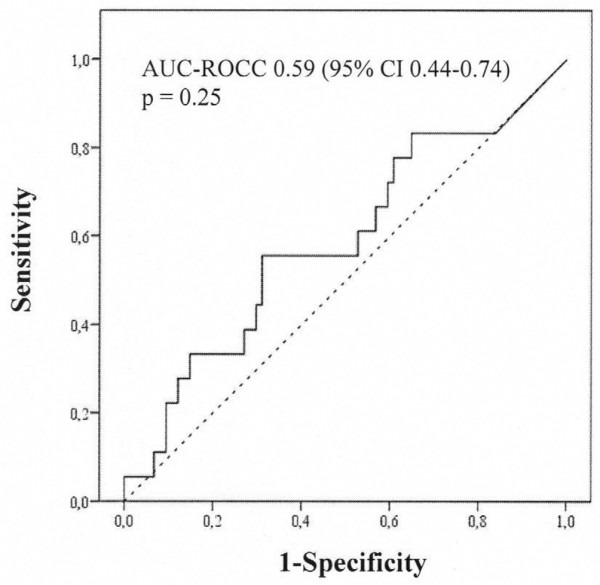
Performance of urinary interleukin-18/urinary creatinine ratio at 24 hours after commencement of cardiopulmonary bypass as a predictor of acute kidney injury according to an increase in serum creatinine of greater than 50% from preoperative to postoperative peak value within 48 hours after commencement of cardiopulmonary bypass (n = 20). AUC-ROCC, area under the curve for the receiver operating characteristic curve; CI, confidence interval.

In this study, we aimed to examine the predictive performance of urinary IL-18 concentration and of urinary IL-18/urinary creatinine ratio on arrival in the intensive care unit (ICU) and at 24 hours after commencement of CPB for the prediction of AKI defined as an increase in serum creatinine of greater than 50% within 48 hours postoperatively (Figures 1, 2, 3, 4). Additional renal outcome measures included AKI defined as a greater than 25% increase in creatinine, changes in creatinine to 72 hours or 5 days, sustained increase of greater than 50%, sustained increase of greater than 25%, or AKI graded according to a variety of RIFLE (Risk of renal dysfunction, Injury to the kidney, Failure of kidney function, Loss of kidney function, and End-stage kidney disease) criteria [14]. We defined 'sustained' as at least two consecutive significantly increased serum creatinine values. In virtually all patients, sampling at 6 hours after commencement of CPB closely corresponded to arrival of the patient in the ICU. We refer to this time point of sampling as 'arrival in ICU'.

### Sampling and measurement of renal function markers

As part of the study protocol, for the measurement of urinary IL-18 and urinary creatinine, we obtained urine preoperatively (immediately before induction of anaesthesia), on arrival in ICU, and at 24 hours after commencement of CPB. Samples were spun at 1,500 *g *for 10 minutes, and the supernatants were stored in equal volumes within 10 minutes at -80°C. We adjusted urinary IL-18 concentration for urinary creatinine concentration (urinary IL-18/urinary creatinine ratio). For the measurement of serum creatinine, we collected blood samples preoperatively and at 24, 48, and 72 hours after commencement of CPB. Urinary IL-18 was measured in duplicate by enzyme-linked immunosorbent assay using matched antibody pairs and recombinant standards (MBL International, Woburn, MA, USA). The inter- and intra-assay coefficients of variation of urinary IL-18 measurement were less than 8%. Serum and urinary creatinine was measured using the Jaffé method (Beckman Coulter SYNCHRON LX System; Beckman Coulter, Inc., Fullerton, CA, USA). The laboratory investigators were blinded to the sample sources and clinical outcomes.

### Data collection

Data collected preoperatively included age, gender, and information on major comorbidities. We collected intraoperative data on type of cardiac surgery and duration of CPB. Finally, we recorded hourly fluid intake and urinary output from start to 24 hours after commencement of CPB (as a urinary catheter was used in all patients during this time). Urinary output was maintained at 0.5 to 1 mL/kg per hour by fluid administration and by using furosemide if necessary.

### Statistical analysis

We assessed data for normal distribution using histograms. When data were normally distributed, we present variables as mean ± standard deviation and used the *t *test for comparison of patients developing AKI with those who did not. When data were not normally distributed, we present variables as median with 25th to 75th percentiles and used the Mann-Whitney *U *test for comparison of both patient groups. When multiple testing occurred, we applied Bonferroni correction. For categorical data, we performed two group comparisons using the chi-square test or Fisher exact test when the expected value was less than 5. Urinary IL-18 was assessed for its ability to detect AKI as defined by nonparametric calculation of the area under the curve for the receiver operating characteristic curve (AUC-ROCC) on arrival in ICU and at 24 hours after commencement of CPB. An AUC-ROCC value of 0.90 to 1.0 indicates excellent, 0.80 to 0.89 good, 0.70 to 0.79 fair, 0.60 to 0.69 poor, and 0.50 to 0.59 no useful performance. Urinary IL-18 concentration was correlated with duration of CPB (Spearman correlation). A two-sided *P *value of 0.05 was considered to be statistically significant. We used SPSS version 15.0 (SPSS Inc., Chicago, IL, USA) and MedCalc version 9.3.9.0 (MedCalc Software, Mariakerke, Belgium).

## Results

We excluded 14 patients and analysed data from 100 adult cardiac surgical patients. None of the patients enrolled received contrast dye within 72 hours preoperatively. Arrival in the ICU was 5.5 ± 1.9 hours after commencement of CPB. Patient characteristics are presented in Table 1. Twenty patients developed AKI. Patients developing AKI were older and were more likely to present with chronic obstructive pulmonary disease, peripheral vascular disease, atrial fibrillation, and arterial hypertension. Within 48 hours postoperatively, the peak serum creatinine concentration was 182.3 ± 56.3 μmol/L in those patients who developed AKI compared with 100.5 ± 27.5 μmol/L in those patients who did not develop AKI (*P *< 0.001). The increase in serum creatinine was sustained for at least 72 hours after CPB, suggesting the presence of intrinsic AKI rather than a prerenal aetiology. In Table 2, we present the concentration of urinary IL-18 comparing patients developing AKI with those who did not. Preoperatively, urinary IL-18 was virtually undetectable. There were no differences in urinary IL-18 on arrival in ICU and at 24 hours postoperatively, even when adjusted for urinary creatinine concentration (Table 2). In Figures 1, 2, 3, 4, we show the AUC-ROCC for the prediction of AKI for urinary IL-18 and urinary IL-18/urinary creatinine ratio on arrival in ICU and at 24 hours, respectively. The predictive performance of urinary IL-18 was not better than chance at either time point (Figures 1 and 2), even after adjustment for urinary creatinine concentration (Figures 3 and 4). Even when using a variety of additional renal outcome measures in an exploratory analysis (>25% increase in creatinine, changes in creatinine to 72 hours or 5 days, sustained increase of greater than 50%, sustained increase of greater than 25%, and a variety of the RIFLE criteria [14]) or the serum creatinine criterion defining AKI according to the network classification [15], the AUC-ROCC for IL-18 was not greater than 0.61 (Tables 3, 4, 5, 6). Serum creatinine measured on arrival in ICU had a higher value (AUC-ROCC 0.69, 95% confidence interval 0.54 to 0.85; *P *= 0.019) to predict AKI (defined as a creatinine increase of greater than 50% within 48 hours postoperatively) than urinary IL-18 at that time of measurement. However, urinary IL-18 correlated with the duration of CPB (Spearman correlation coefficient 0.47; *P *< 0.001).

**Table 1:** Preoperative characteristics of and intraoperative interventions in patients

Characteristics	AKI (n = 20)	No AKI (n = 80)	*P *value
Demographic data			
Age, years	75.3 ± 4.1	68.1 ± 9.7	<0.001
Female, number (percentage)	6 (30.0)	33 (41.0)	0.36
Body mass index	29.7 ± 4.3	27.4 ± 4.5	0.045
Comorbidities			
Preoperative serum creatinine, μmol/L	96.1 ± 31.6	89.4 ± 24.0	0.30
Preoperative estimated glomerular filtration rate < 60 mL/minute, number (percentage)	8 (40.0)	19 (23.8)	0.14
Left ventricular dysfunction (ejection fraction < 40%), number (percentage)	7 (35.0)	18 (22.5)	0.25
Arterial hypertension, number (percentage)	20 (100.0)	64 (80.0)	0.029
Hypercholesterolemia, number (percentage)	16 (80.0)	46 (57.5)	0.06
Atrial fibrillation, number (percentage)	10 (50.0)	16 (20.0)	0.006
Myocardial infarction (within 6 months preoperatively), number (percentage)	7 (35.0)	13 (16.3)	0.06
Chronic obstructive pulmonary disease, number (percentage)	6 (30.0)	8 (10.0)	0.021
Peripheral vascular disease, number (percentage)	4 (20.0)	2 (2.5)	0.013
Diabetes mellitus, number (percentage)^a^	8 (40.0)	20 (25.0)	0.18
Intervention			
Coronary artery bypass grafting surgery, number (percentage)	7 (35.0)	18 (22.5)	0.25
Valvular surgery, number (percentage)	7 (35.0)	45 (56.3)	0.09
Simultaneous coronary revascularisation and valve surgery, number (percentage)	6 (30.0)	11 (13.8)	0.08
Duration of cardiopulmonary bypass (minutes)	147.0 ± 31.6	136.0 ± 41.0	0.20
Fluid intake, 0–24 hours, mL^b^	5,500 ± 1,900	5,150 ± 1,250	0.44
Urine output, 0–24 hours, mL	3,350 ± 1,050	3,990 ± 1,500	0.034
Furosemide dose, 0–24 hours, mg	133.9 ± 168.7	30.9 ± 34.8	<0.001
Outcomes			
Need for renal replacement therapy or died during hospital stay, number (percentage)	4 (20.0)	1 (1.3)	0.005
Ventilation, days	2.4 ± 3.1	0.9 ± 0.8	0.010
Length of stay in the intensive care unit, days	6.3 ± 7.1	2.3 ± 2.1	0.012
Length of stay in hospital, days	16.2 ± 13.8	9.5 ± 4.7	0.026

^a^Diabetes mellitus includes patients on antidiabetic medication and on insulin. ^b^Crystalloids, colloids, and oral fluids from the commencement of cardiopulmonary bypass to 24 hours thereafter. AKI, acute kidney injury.

**Table 2:** Postoperative urinary interleukin-18 (IL-18) concentration

Urinary IL-18	AKI (n = 20)	No AKI (n = 80)	*P *value
Unadjusted values			
Preoperative, pg/mL	0 (0–25)	0 (0–44)	0.28
On arrival in the intensive care unit, pg/mL	168 (19–736)	104 (36–292)	0.70
At 24 hours postoperatively, pg/mL	195 (54–537)	165 (42–288)	0.47
Values adjusted for urinary creatinine			
Preoperative, pg/mg	0 (0–12)	0 (0–32)	0.32
On arrival in the intensive care unit, pg/mg	332 (44–1,789)	233 (86–531)	0.57
At 24 hours postoperatively, pg/mg	190 (75–478)	124 (41–245)	0.25

Values denoting median and 25th to 75th percentiles. *P *values indicate Mann-Whitney *U *test. AKI, acute kidney injury.

**Table 3:** Performance characteristics of urinary interleukin-18 (IL-18) according to different renal outcome definitions

Variables	AUC-ROCC (95% CI)	*P *value
Increase in serum creatinine > 25% within 24 hours postoperatively		
Urinary IL-18 on arrival in the intensive care unit	0.58 (0.46–0.71)	0.19
Urinary IL-18 at 24 hours after cardiopulmonary bypass	0.56 (0.43–0.69)	0.34
Increase in serum creatinine > 50% within 24 hours postoperatively		
Urinary IL-18 on arrival in the intensive care unit	0.56 (0.38–0.74)	0.51
Urinary IL-18 at 24 hours after cardiopulmonary bypass	0.57 (0.40–0.73)	0.45
Increase in serum creatinine > 25% within 48 hours postoperatively		
Urinary IL-18 on arrival in the intensive care unit	0.59 (0.47–0.71)	0.14
Urinary IL-18 at 24 hours after cardiopulmonary bypass	0.56 (0.43–0.68)	0.36
Increase in serum creatinine > 50% within 48 hours postoperatively		
Urinary IL-18 on arrival in the intensive care unit	0.53 (0.38–0.68)	0.70
Urinary IL-18 at 24 hours after cardiopulmonary bypass	0.55 (0.40–0.71)	0.48
Increase in serum creatinine > 25% within 72 hours postoperatively		
Urinary IL-18 on arrival in the intensive care unit	0.60 (0.39–0.71)	0.63
Urinary IL-18 at 24 hours after cardiopulmonary bypass	0.58 (0.44–0.72)	0.55
Increase in serum creatinine > 50% within 72 hours postoperatively		
Urinary IL-18 on arrival in the intensive care unit	0.58 (0.36–0.66)	0.85
Urinary IL-18 at 24 hours after cardiopulmonary bypass	0.55 (0.41–0.69)	0.50
Increase in serum creatinine > 25% within 120 hours postoperatively		
Urinary IL-18 on arrival in the intensive care unit	0.60 (0.48–0.72)	0.10
Urinary IL-18 at 24 hours after cardiopulmonary bypass	0.59 (0.47–0.71)	0.14
Increase in serum creatinine > 50% within 120 hours postoperatively		
Urinary IL-18 on arrival in the intensive care unit	0.52 (0.38–0.67)	0.78
Urinary IL-18 at 24 hours after cardiopulmonary bypass	0.56 (0.43–0.70)	0.38

AUC-ROCC, area under the curve for the receiver operating characteristic curve; CI, confidence interval.

**Table 4:** Performance characteristics of urinary interleukin-18 (IL-18) according to different RIFLE criteria

Variables	AUC-ROCC (95% CI)	*P *value
RIFLE R or worse (R: n = 31 + I: n = 13 + F: n = 6)		
Urinary IL-18 on arrival in the intensive care unit	0.61 (0.48–0.74)	0.12
Urinary IL-18 at 24 hours after cardiopulmonary bypass	0.57 (0.43–0.71)	0.30
RIFLE I or worse (I: n = 13 + F: n = 6)		
Urinary IL-18 on arrival in the intensive care unit	0.52 (0.38–0.68)	0.35
Urinary IL-18 at 24 hours after cardiopulmonary bypass	0.58 (0.43–0.73)	0.28

For RIFLE classes [14], serum creatinine increases within 120 hours postoperatively and urinary output during the first 24 hours postoperatively. AUC-ROCC, area under the curve for the receiver operating characteristic curve; CI, confidence interval; RIFLE, Risk of renal dysfunction, Injury to the kidney, Failure of kidney function, Loss of kidney function, and End-stage kidney disease; RIFLE F, RIFLE Failure; RIFLE I, RIFLE Injury; RIFLE R, RIFLE Risk.

**Table 5:** Performance characteristics of urinary interleukin-18 (IL-18) according to different network criteria of acute kidney injury

Variables	AUC-ROCC (95% CI)	*P *value
AKIN stage 1 or worse (n = 32)		
Urinary IL-18 on arrival in the intensive care unit	0.48 (0.35–0.61)	0.80
Urinary IL-18 at 24 hours after cardiopulmonary bypass	0.59 (0.46–0.72)	0.15

Acute kidney injury defined according to the network criteria [15] with serum creatinine increase of greater than 26.4 μmol/L or greater than 0.3 mg/dL within 48 hours postoperatively. AKIN, Acute Kidney Injury Network; AUC-ROCC, area under the curve for the receiver operating characteristic curve; CI, confidence interval.

**Table 6:** Performance characteristics of urinary interleukin-18 (IL-18) to predict the development of sustained acute kidney injury

Variables	AUC-ROCC (95% CI)	*P *value
Increase in serum creatinine > 25% within 48 hours postoperatively		
Urinary IL-18 on arrival in the intensive care unit	0.58 (0.46–0.71)	0.19
Urinary IL-18 at 24 hours after cardiopulmonary bypass	0.56 (0.43–0.69)	0.43
Increase in serum creatinine > 50% within 48 hours postoperatively		
Urinary IL-18 on arrival in the intensive care unit	0.56 (0.38–0.74)	0.51
Urinary IL-18 at 24 hours after cardiopulmonary bypass	0.57 (0.40–0.73)	0.45
Increase in serum creatinine > 25% within 120 hours postoperatively		
Urinary IL-18 on arrival in the intensive care unit	0.59 (0.47–0.71)	0.14
Urinary IL-18 at 24 hours after cardiopulmonary bypass	0.56 (0.43–0.68)	0.36
Increase in serum creatinine > 50% within 120 hours postoperatively		
Urinary IL-18 on arrival in the intensive care unit	0.51 (0.34–0.67)	0.93
Urinary IL-18 at 24 hours after cardiopulmonary bypass	0.57 (0.42–0.73)	0.36
RIFLE R or worse (R: n = 31 + I: n = 13 + F: n = 6)		
Urinary IL-18 on arrival in the intensive care unit	0.61 (0.48–0.74)	0.12
Urinary IL-18 at 24 hours after cardiopulmonary bypass	0.57 (0.43–0.71)	0.29
RIFLE I or worse (I: n = 13 + F: n = 6)		
Urinary IL-18 on arrival in the intensive care unit	0.52 (0.37–0.67)	0.30
Urinary IL-18 at 24 hours after cardiopulmonary bypass	0.58 (0.43–0.73)	0.28

Acute kidney injury is defined as at least two consecutive significantly increased serum creatinine values. For RIFLE classes, serum creatinine increases within 120 hours postoperatively and urinary output during the first 24 hours postoperatively. AUC-ROCC, area under the curve for the receiver operating characteristic curve; CI, confidence interval; RIFLE, Risk of renal dysfunction, Injury to the kidney, Failure of kidney function, Loss of kidney function, and End-stage kidney disease; RIFLE F, RIFLE Failure; RIFLE I, RIFLE Injury; RIFLE R, RIFLE Risk.

## Discussion

In this prospective observational cohort study of 100 adult cardiac surgical patients, we analysed the predictive performance for AKI of early postoperative urinary IL-18 measurement on arrival in ICU and at 24 hours after commencement of CPB. In this setting, urinary IL-18 did not appear to be a useful biomarker for the prediction of AKI. AKI after CPB is associated with adverse hospital outcomes. Patients who develop AKI have increased morbidity and hospital mortality [1,2]. Early measurement of serum creatinine is considered to be of limited value in the prediction of AKI because it typically increases only when the glomerular filtration rate has decreased to less than 50% of normal [7,15]. Urinary IL-18, however, appears to have value in the prediction of AKI [6,10-13]. It has been demonstrated in renal tubular epithelial cells and in activated macrophages [8,16]. However, urinary IL-18, a promising renal biomarker for the early diagnosis of AKI in children undergoing cardiac surgery, has not yet been investigated in adult cardiac surgical patients.

The good predictive performance of urinary IL-18 for the diagnosis of AKI in a recent study of paediatric cardiac surgical patients [13] was not confirmed for adults in this study. However, in paediatric cardiac surgical patients, comorbidities do not play such a major and confounding role compared with adults. Adult cardiac surgical patients often present with age-related comorbidities and with diminished renal reserve. Accordingly, observations made in paediatric populations may not apply to adult patients. This appears to be the case for urinary IL-18.

Our negative findings are in accordance with a previous study investigating the value of urinary IL-18 in the prediction of AKI following exposure to contrast for coronary intervention in adult patients [9]. While it is conceivable that the very early peak urinary IL-18 concentration was missed in this study [9] – even when measured (as we did) at an early stage (6 hours after the injurious event to the kidney commenced) – urinary IL-18 was not able to predict AKI. It is nonetheless important to note that patients undergoing CPB who did not develop AKI had elevated postoperative urinary IL-18 concentrations compared with preoperative values. There are at least three possible explanations: (a) all patients undergoing CPB suffer mild AKI, which resolves quickly and does not lead to any elevation in serum creatinine; or (b) urinary IL-18 increases independently of kidney injury, due to nonrenal factors such as systemic inflammation; or (c) both explanations apply. Indeed, we found that duration of CPB correlated with postoperative urinary IL-18. On the basis of our findings, increases in urinary IL-18 may indicate renal epithelial tubular cell injury in adult cardiac surgical patients, which is not severe enough to predictably lead to loss of glomerular filtration rate. Thus, urinary IL-18 is potentially a nonspecific marker of inflammation and not a specific marker for AKI. Indeed, IL-18 is released by both renal tubular cells and macrophages or blood monocytes and is then filtered through the glomerular basal membrane. The latter source, however, is believed to represent the main origin of IL-18 in adults [8]. If this were true, it would potentially confound the value of urinary IL-18 as a specific renal biomarker and at least partly explain our observations. In children, release of IL-18 may proceed primarily through the kidney rather than immune cells, thus explaining the better predictive ability of IL-18. Irrespective of the possible explanation, our observations highlight the need to study the value of renal biomarkers in adult cardiac surgical patients separately.

This is a single-centre study and its findings need to be confirmed or refuted in other centres. While it is conceivable that a larger sample size or more frequent measurement might have generated statistically significant findings on urinary IL-18, the study sample size is sufficient for the detection of a major and clinically relevant predictive performance of this renal biomarker. Although we enrolled a patient population with a typical mix of cardiac surgical procedures, it is possible that urinary IL-18 would be of greater value in patients at increased risk of developing AKI. In the absence of adjudication of aetiology, the sustained increase in serum creatinine may exclude, at least in part, haemodynamic/prerenal causes of AKI, for which no increase in urinary IL-18 would be expected. Also, worse outcome in patients developing AKI (as defined) compared with those who did not develop AKI points to intrinsic rather than prerenal azotaemia. We consider that additional novel biomarkers with increased predictive performance are still needed for more precise and earlier prediction of AKI after adult cardiac surgery.

## Conclusion

This is the first study to test the value of urinary IL-18 as a predictor of AKI following adult cardiac surgery with CPB. Our findings suggest that, although urinary IL-18 may be a marker of CPB-associated inflammation, it is not useful in identifying patients who will subsequently develop AKI after adult cardiac surgery.

## Key messages

• Urinary interleukin-18 (IL-18) measured on arrival in the intensive care unit and at 24 hours postoperatively does not predict acute kidney injury in adult cardiac surgical patients.

• Urinary IL-18 appears instead to be a marker of inflammation in this setting.

## Abbreviations

AKI = acute kidney injury; AUC-ROCC = area under the curve for the receiver operating characteristic curve; CPB = cardiopulmonary bypass; ICU = intensive care unit; IL-18 = interleukin-18; RIFLE = Risk of renal dysfunction, Injury to the kidney, Failure of kidney function, Loss of kidney function, and End-stage kidney disease.

## Competing interests

The authors declare that they have no competing interests.

## Authors' contributions

MH and AH-F participated in the design of the study, performance of the statistical analysis, and drafting of the manuscript. RB conceived of the study and participated in the design of the study, performance of the statistical analysis, and drafting of the manuscript. PD carried out the urinary IL-18 measurements. DS participated in the design and coordination of the study and helped to draft the manuscript. All authors read and approved the final manuscript.
